# The Geriatric Acute and Post-Acute Fall Prevention Intervention (GAPcare) II to Assess the Use of the Apple Watch in Older Emergency Department Patients With Falls: Protocol for a Mixed Methods Study

**DOI:** 10.2196/24455

**Published:** 2021-04-01

**Authors:** Daniel H Strauss, Natalie M Davoodi, Margaret Healy, Christopher L Metts, Roland C Merchant, Swechya Banskota, Elizabeth M Goldberg

**Affiliations:** 1 Alpert Medical School of Brown University Providence, RI United States; 2 Brown University School of Public Health Providence, RI United States; 3 Rhode Island Hospital Providence, RI United States; 4 Department of Pathology and Laboratory Medicine Medical University of South Carolina Charleston, SC United States; 5 Department of Emergency Medicine Brigham and Women’s Hospital Harvard Medical School Boston, MA United States; 6 Department of Emergency Medicine Alpert Medical School of Brown University Providence, RI United States

**Keywords:** fall intervention, geriatric care, Apple Watch, wearable technology

## Abstract

**Background:**

Falls are a common problem among older adults that lead to injury, emergency department (ED) visits, and institutionalization. The Apple Watch can detect falls and alert caregivers and clinicians that help is needed; the device could also be used to objectively collect data on gait, fitness, and falls as part of clinical trials. However, little is known about the ease of use of this technology among older adult ED patients, a population at high risk of recurrent falls.

**Objective:**

The goal of this study—the Geriatric Acute and Post-Acute Fall Prevention Intervention (GAPcare) II—is to examine the feasibility, acceptability, and usability of the Apple Watch Series 4 paired with the iPhone and our research app Rhode Island FitTest (RIFitTest) among older adult ED patients seeking care for falls.

**Methods:**

We will conduct field-testing with older adult ED patients (n=25) who sustained a fall and their caregivers (n=5) to determine whether they can use the Apple Watch, iPhone, and app either (1) continuously or (2) periodically, with or without telephone assistance from the research staff, to assess gait, fitness, and/or falls over time. During the initial encounter, participants will receive training in the Apple Watch, iPhone, and our research app. They will receive an illustrated training manual and a number to call if they have questions about the research protocol or device usage. Participants will complete surveys and cognitive and motor assessments on the app during the study period. At the conclusion of the study, we will solicit participant feedback through semistructured interviews. Qualitative data will be summarized using framework matrix analyses. Sensor and survey response data will be analyzed using descriptive statistics.

**Results:**

Recruitment began in December 2019 and was on pause from April 2020 until September 2020 due to the COVID-19 pandemic. Study recruitment will continue until 30 participants are enrolled. This study has been approved by the Rhode Island Hospital Institutional Review Board (approval 1400781-16).

**Conclusions:**

GAPcare II will provide insights into the feasibility, acceptability, and usability of the Apple Watch, iPhone, and the RIFitTest app in the population most likely to benefit from the technology: older adults at high risk of recurrent falls. In the future, wearables could be used as part of fall prevention interventions to prevent injury before it occurs.

**Trial Registration:**

ClinicalTrials.gov NCT04304495; https://clinicaltrials.gov/ct2/show/NCT04304495

**International Registered Report Identifier (IRRID):**

DERR1-10.2196/24455

## Introduction

Falls are the leading cause of emergency department (ED) visits for injuries in older adults aged 65 and older [1,2]. Each year in the United States, 28.7% of older adults sustain a fall, resulting in 2.8 million annual ED visits for geriatric falls [3]. The risk of recurrent falls is particularly high immediately after the ED visit [4]; 31% of community-dwelling older adults in the United States fall again within 6 months, and 62% of these falls cause serious injury [5,6]. Technology-based interventions initiated immediately after the ED visit could be useful to prevent falls, if they are embraced by older adults.

Wearable technology, while conventionally targeted toward a younger population, has shown promise in older adults. One recent prospective study with 95 community-dwelling older adults found as many as 91% of participants rated a wearable watch as acceptable and easy to use [7]. Additionally, the Apple Heart Study, launched in 2017, recruited over 24,000 older adults to monitor their heart rhythm for atrial fibrillation using the Apple Watch and an iPhone app [8], indicating that this population can use these devices. Furthermore, a recent systematic review showed that exercise, compared to educational or environmental interventions, significantly reduced falls in older adults, although increased walking alone did not decrease falls [9]. Wrist-worn technologies may increase daily step counts [10] and encourage exercise tracking, which could facilitate fall prevention. Fitness tracking has the additional psychological benefit of allowing individuals to have control of their subjective well-being, how people experience and evaluate the quality of their lives [11].The prospect of these devices for fall-related measures in older adults remains a relatively untapped area for application of this technology.

The Apple Watch (Apple Inc) is a wearable device with built-in sensors that can detect falls automatically using its accelerometer and gyroscope sensors. Unlike traditional fall alert pendants, the Apple Watch alerts designated contacts and emergency medical services even if the individual is unconscious or immobile. The Apple Watch is particularly well-suited as a research tool because fall occurrences and other sensor data (eg, heart rate and step count) are recorded in HealthKit, a developer application programming interface included in the iPhone operating system (iOS) [12]. Additionally, Apple provides developers with open-source code for cognitive and motor tests called *active tasks*, which allow participants to perform assessments independently at home with the help of visual, tactile, and auditory prompts generated by the iPhone. Results of these assessments, as well as fall occurrences and other HealthKit-collected data, are recorded into our novel research app Rhode Island FitTest (RIFitTest) and transmitted to Research Electronic Data Capture (REDCap) [13] in real time for monitoring and analysis by the research team.

We used the Unified Theory of Acceptance and Use of Technology (UTAUT) model as a conceptual framework when developing our protocol. The UTAUT model, which is a combination of eight prominent theories—the Theory of Reasoned Action, Innovation Diffusion Theory, the Theory of Planned Behavior (TPB), the Technology Acceptance Model (TAM), the combined TAM-TPB, Social Cognitive Theory, the Motivational Model, and the Model of Personal Computer Utilization—has been extensively tested across multiple disciplines and has been validated as an effective tool to assess acceptance of health-related technology [14]. In the UTAUT model, four constructs play a significant role as determinants of user acceptance and usage of technology: (1) performance expectancy, (2) effort expectancy, (3) self-efficacy, and (4) facilitating conditions.

In this mixed methods study—the Geriatric Acute and Post-Acute Fall Prevention Intervention (GAPcare) II—we will conduct field-testing with older adult ED patients, with and without cognitive impairment, who present to the ED with a fall within the last 7 days, as well as with their caregivers, to assess the feasibility, acceptability, and usability of the Apple Watch, iPhone, and the RIFitTest app to assess gait, fitness, and/or subsequent falls.

## Methods

### Study Design

Because we are interested in reducing recurrent falls among older adult ED patients, we designed a mixed methods study to collect perspectives from older adults and their caregivers through semistructured interviews (ie, qualitative data) and information about falls and fall risk factors prospectively (ie, quantitative data). Fall risk factors include impairment in cognition, impairment in mobility, fear of falling, and heart rate abnormalities, all of which can be measured by wearable device sensors or through survey questions on mobile devices. This study was registered at ClinicalTrials.gov (NCT04304495).

### Field-Testing Nature of the Study

Older adults face unique challenges with using mobile devices, and many studies have shown that interest in using wearables often wanes after initial recruitment [15]. Therefore, we decided we needed to first field-test our protocol among older adults to ensure they would express interest in participating, be able to learn how to use the technology, and stay continuously engaged in the study. A field test is a type of testing that evaluates technology performance in real-world settings. This allows for an enhanced understanding of how the target end user experiences the product and can lead to design optimization and insights into challenges that must still be overcome.

### Planning Phase

During the planning phase, we assembled experts in digital health, qualitative research, geriatrics, neurocognitive assessments, and clinical research. This team advised the principal investigator (EG) on the protocol, outcome assessment, and implementation of the study. We also programmed our survey questions in REDCap, created the app, and tested the app among our research staff during this time.

### Information Technology Solution

To collect the sensor and survey data generated on the Apple Watch and to transfer the data to our secure server, we needed to develop an iOS app. We engaged one of the study authors (CM) to assist with creating the app for the study using his third-party platform, status/post [16]. Status/post integrates REDCap and ResearchKit and allows researchers to design the app in the same way questionnaires are designed in REDCap, reducing costs and turnaround time because a developer is not necessary.

### RIFitTest Study App

This app, when paired with the Apple Watch and the user’s iPhone, collects sensor-obtained data *passively*. Users can also enter data *actively* using their phones. Several steps are required to create the research app, RIFitTest:

1. Enter survey questions and answers into REDCap data collection software.
2. Choose Apple HealthKit measures (eg, heart rate and steps) and enter Apple Inc open-source code into the field notes of REDCap to extract sensor data from the watch and store it in REDCap.
3. Select Apple ResearchKit *active tasks* relevant to the study and enter Apple Inc open-source code into the field notes of REDCap.
4. Use status/post to schedule notifications for participants, create participant usernames and passwords, and monitor participant activity.
5. Post app on Apple App Store to allow participants to download the app onto their iPhones.

After creation of the app, we will pilot-test it among the research staff and make changes to improve the usability for older adults (eg, increase font size and choose the color of the app to enhance readability). Only participants who complete the consent process will be able to download the study app. When installing the RIFitTest app, participants will enter a username and password created by the study team and will permit the app to access each category of data stored in Apple HealthKit (eg, heart rate and step count).

### Data Security, Transmission, and Storage

Data from the device will be pushed to the Health Insurance Portability and Accountability Act (HIPAA)-compliant REDCap program when participants connect to a Wi-Fi network, or the data will be added to the REDCap program manually during follow-up with the research team. We will use an application programming interface to ensure data can be viewed in real time on the REDCap program. Data transmission is encrypted with Secure Sockets Layer. Similar to other iOS-based apps, the RIFitTest app is “sandboxed,” meaning that no other apps can gather data collected by the study app. The app is password protected, which prevents user data from being revealed if the device is stolen.

### Study Protocol

#### Setting

The study will be conducted at two EDs in Providence, Rhode Island: Rhode Island Hospital (RIH), a tertiary-care hospital, and The Miriam Hospital (TMH), an academic community hospital. RIH is the only Level I trauma center in the state, while patients at TMH are primarily community-dwelling older adults.

#### Population

We will recruit 30 participants. These participants will be recruited into six groups with 5 participants each from two different cohorts: cognitively intact and cognitively impaired. Cognitively intact participants will include 20 patients: ages 65-69 (n=5), ages 70-74 (n=5), ages 75-79 (n=5), and ages 80-84 (n=5). Cognitively impaired participants aged 65 years old and older (n=5) will be enrolled with their caregivers (n=5). Caregivers will complete the same tasks as the patient and are encouraged to assist the patient in using the technology. Their perspectives are important for understanding how the technology can be used for communication and to better understand facilitators and barriers of the technology. Quota sampling will be employed to ensure study participants reflect the racial and ethnic diversity of Rhode Island.

#### Eligibility

Eligible participants will be noninstitutionalized, community-dwelling older adults aged 65 years and older who are English-speaking and present to the ED after a fall within the past 7 days. Patients with cognitive impairment, as measured by a score of less than 4 on the Six-Item Screener [17], must have a legally authorized representative available to give informed consent. The patient’s ED physician must intend to discharge the patient after their initial evaluation either to their home or to an assisted living or rehabilitation center. Patients with falls due to syncope, an externally applied force, or critical illness (eg, stroke) will be excluded. Patients who present with altered mental status (eg, intoxicated or agitated), have injuries that prevent mobilization (eg, pelvic or lower extremity fractures), are undomiciled, have allergies to any wearable device component, are unable or unwilling to wear an Apple Watch at home, or have advanced cancer and/or are in hospice care also will be excluded.

#### Recruitment and Enrollment

Eligible patients will be approached by a member of the research team and asked if they are interested in participating. If interested, participants will provide written informed consent. Those who consent will answer questions on prior technology use, demographic characteristics, previous falls, and their health history. Participants will receive an Apple Watch Series 4 and an iPhone 7—if they do not already own these devices—for the duration of the study. Participants will be able to use their own Apple Watch and iPhone for the study.

#### Apple Watch Specifications and Deployment

For this study, each participant will use the Apple Watch Series 4 (GPS), manufactured by Quanta Computer Compal Electronics. We will use the Apple Watch with the larger 44-mm watch face and Velcro wrist strap to make it easier to use for older adults with vision impairment or those lacking fine motor skills.

#### ED Procedures

The following steps will be taken for device setup *prior* to patient approach:

1. Charge and pair the Apple Watch and iPhone via Bluetooth; for participants with their own devices, pairing will occur in real time.
2. Maximize font and icon size on both devices to enhance readability.
3. Set brightness at its maximum to improve readability.
4. Delete irrelevant content and minimize notifications (eg, remove and mute unnecessary apps and notifications, respectively) to limit distractions.
5. Download the RIFitTest app from the Apple App Store onto the iPhone.
6. Add the study principal investigator and an emergency contact to the contact list to receive fall alert notifications.

Training in the use of the iPhone, Apple Watch, and RIFitTest app as well as study-related activities will also be provided, including the following:

1. iPhone and Apple Watch setup instructions, including how to turn the device on and off, unlock and set a password, and charge both devices.
2. Real-time demonstration of *active tasks* accessed through the study app.
3. A demonstration of how to log a fall and/or injury through the app.
4. Education in the research protocol, including when to use the Apple Watch (ie, continuously, including while bathing and participating in water activities), nightly charging of iPhone, mealtime charging of the Apple Watch, daily completion of fall surveys, and weekly completion of *active tasks* on the RIFitTest app.
5. Orientation to the study materials: file, business card with hotline for assistance, technology manual (see Multimedia Appendix 1), calendar of assessment timing, technology agreement, and consent form.

**Figure 1:**
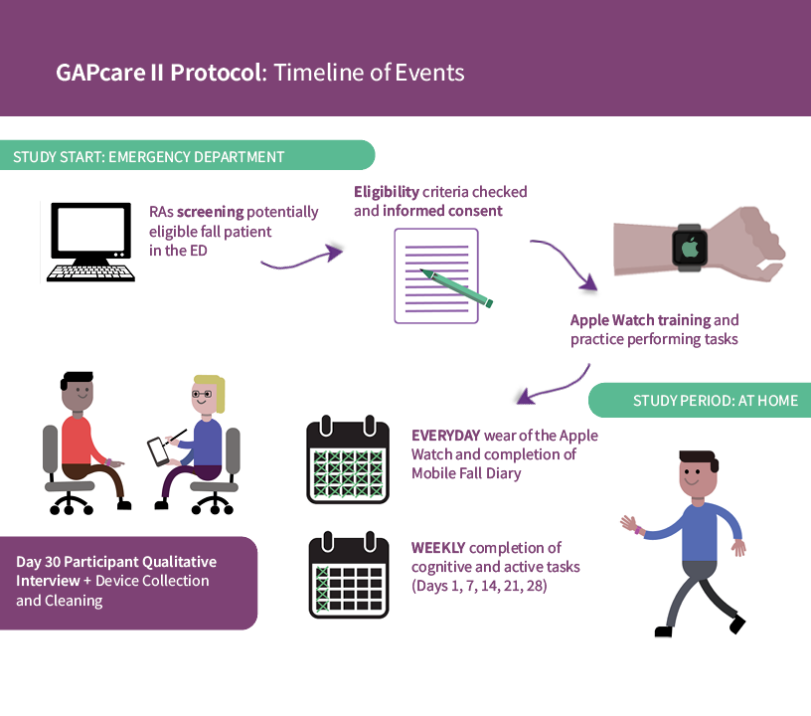
Timeline of study duration. ED: emergency department; GAPcare II: Geriatric Acute and Post-Acute Fall Prevention Intervention II; RA: research assistant.

Figure 1 illustrates a timeline of the study that will be explained to participants.

#### Follow-up Procedures

Study participation will last for 30 days or until the participant is no longer able or willing to participate. All participants will be contacted by research staff on days 3, 8, 15, and 22 to remind them to complete the steps in the study protocol and to answer questions. Participants will take part in a 30-minute semistructured interview at the completion of the study period. The interview will be conducted by the principal investigator and her staff. The intent of the interview will be to record participants’ experience with the study as well as to provide information on the feasibility, usability, and acceptability of the Apple Watch, iPhone, and RIFitTest app. Participant feedback about the quality of the intervention materials will also be solicited. The information collected in the interview will be used to improve the app and training procedures in preparation of the subsequent clinical trial.

#### Measures

There are five data sources that will contribute data for this field test: research staff–administered surveys, qualitative interviews with patients and caregivers, Apple Watch sensor-obtained measures, user-completed iPhone surveys (ie, a digital fall diary, fall efficacy survey, and a usability survey), and user-completed *active tasks* (ie, guided motor and cognitive assessments). Specific measures are summarized in Table 1.

**Table 1:** Study instruments, surveys, and active tasks.

Instrument			Description	Administration time
**Process evaluation**				
	**Research staff–administered survey**			
		Screening, eligibility, and retention	This survey will record how many patients were screened, agreed to participate, were recruited, received intended treatment, and were retained.	Baseline and 30 days
**Outcome evaluation**				
	**Research staff–administered surveys and interviews**			
		Enrollment questionnaire	Demographic characteristics Prior fall history, comorbidities, emergency department index visit fall circumstances, and injuries	Baseline; <5 minutes
		Six-Item Screener [17]	6-point questionnaire to measure cognitive impairment for study screening; <4 indicates high risk for cognitive impairment	Baseline; 2 minutes
		Qualitative interviews	These interviews will track follow-up phone sessions with research staff. Interviews record participants’ experience with the study while also establishing an understanding of the usability of both the Apple Watch and RIFitTest^a^ app for older adults. The timing and length of each session and barriers to attendance are recorded.	Days 3, 8, 15, 22, and 30
	**Apple Watch sensor-obtained measures**			
		Apple Watch measures (ie, accelerometer, gyroscope, and physiological sensors)	Gait and fitness (ie, time spent walking, standing, and climbing steps; 4-meter walk gait speed test; and resting heart rate) Data will include cognitive and active task performance, including cadence and walking speed related to their gait and balance and Stroop test. Performance reports will be generated for each participant.	Baseline ± continuously (days 1 to 30)
	**User-completed iPhone surveys**			
		Fall diary	Daily app-based entry that will ask participants if they have fallen	Days 1 to 30
		Fall-efficacy survey	Measure of fear of falling, which increases fall risk	Days 1 and 28
		Usability survey	This survey will record each participant’s experience with the app, as well as with the active and cognitive tasks.	Days 7 and 28
	**User-completed *active tasks* [12] on the iPhone**			
		Gait and balance test	Active task; measure of 20 steps of walking in one direction, returning, and then standing still for 30 seconds	Days 1, 7, 14, 21, and 28
		Timed walk	Measure of a walking distance of 109 yards in a straight line as quickly as possible	Days 1, 7, 14, 21, and 28
		Stroop test	Measure of ability to quickly identify color of text	Days 1, 7, 14, 21, and 28
		Reaction time test	Active task; measure of speed at clicking a button when shown	Days 1, 7, 14, 21, and 28
		Trail-making test	Active task; measure of ability to follow an alphanumeric sequence (1...A...2...B..., etc)	Days 1, 7, 14, 21, and 28

^a^RIFitTest: Rhode Island FitTest.

### Qualitative Interviews

#### Interview Procedure

Study staff will conduct interviews with both the participants and their caregivers, when applicable. They will use an interview guide containing semistructured questions with follow-up questions and probes to explore participants’ responses.

#### Recording

Interviews will be transcribed and deidentified. Transcripts will be corrected for accuracy as needed using the audio recording.

#### Analysis

We will use *framework analysis*—a qualitative analysis technique, in which investigators summarize content within categories into charts after transcription [18,19]—as it is particularly well-suited to generate recommendations within a limited time period, so as to inform the subsequent clinical trial. Another benefit of the framework method is the emphasis on transparency in data analysis and the links between the stages of the analysis [19]. Framework methods include summarizing qualitative data into a matrix or spreadsheet where data are entered by codes (columns) and cases (rows) [20]. Specifically, we will take the following steps:

1. Read and re-read the transcripts to note initial observations; iteratively search for common themes, subthemes, and patterns across participant responses; and develop a coding matrix and assign data to the themes and categories in the coding matrix.
2. Review themes in relation to the extracts and entire data set; identify associations between the themes until a “whole picture” is apparent.
3. Select representative quotes from the interviews to illustrate the themes.
4. Record ideas about emerging themes in an ongoing audit trail.
5. Prepare the analytic narrative and contextualize it using existing research on this topic.

#### Debriefing

Interviewers will complete a debriefing form after the interview to document the following: (1) tone of interview, (2) agenda adherence, (3) interview description, (4) major themes, (5) lessons learned, (6) question strategies, and (7) saturation.

#### Poststage Interview Content

The following three study aims will be addressed through qualitative data collected during the poststage interview:

1. Feasibility: (1) ability to carry out device charging, application, and manipulation; (2) barriers to daily use; and (3) responses to specific planned components of the intervention.
2. Acceptability: receptivity to and concerns about device use.
3. Usability: (1) prior experiences with wearables; (2) trajectories of use, including reasons for continuation or cessation of use; (3) quality of in-person and telephone device support and training; (4) connectivity to smartphone and Wi-Fi, if desired; (5) caregiver experiences; and (6) patient and caregiver’s perceived needs and preferred method of use.

Interview questions will be grounded in the four constructs of the UTAUT model.

### Sensor and Survey Data Collection and Analysis

Continuous data collected by the Apple Watch will be sourced from the accelerometer, gyroscope, heart rate monitor, and GPS sensors. Episodic data that will be collected include electronic health record data, fall dairy, Apple Watch measures, RIFitTest app data*,* and the fall-efficacy scale, as outlined in Table 1. This combined data set will then be analyzed descriptively and using longitudinal methods, as appropriate.

Descriptive statistics will be used to measure the following key parameters of feasibility of recruitment, enrollment, participation, and retention [21]: number of patients screened, eligible, and recruited; time required to recruit; number of patients unable to provide consent; number of dropouts; and number of patients with refusal and retention at each follow-up. Frequencies, proportions, rates, means and medians, standard deviations, and other measures of variability will be used to report on these feasibility measures.

Descriptive statistics will also be used to assess the following parameters of acceptability: the fall diary completion rates, days the Apple Watch was worn, and any malfunctions of the Apple Watch or app. We will use the Apple Watch–obtained data to determine the association between gait and fitness measures (eg, step count, stairs climbed, and resting heart rate) and reported falls. To account for the longitudinal nature of this data and within-person correlation, we will use generalized estimating equations or generalized linear models [22].

### Privacy and Data Storage

The Apple Watch and iPhone will be password protected. All devices will be cleaned following hospital procedures and the stored data will be erased upon return of these devices at the end of the participants’ involvement in the study.

## Results

Recruitment began in December 2019 and was on pause from April 2020 until September 2020 due to the COVID-19 pandemic. Funding for this work includes a K76 grant awarded by the National Institute on Aging (NIA), via the Paul B Beeson Emerging Leaders Career Development Award in Aging (grant K76AG059983), for the period of September 1, 2019, to September 1, 2024. This study has been approved by the RIH Institutional Review Board (approval 1400781-16).

## Discussion

### Overview

GAPcare II will field-test the Apple Watch, iPhone, and our novel research app RIFitTest among older adults. Our study may open new research horizons by providing data on how to implement sensor-based assessment in real-world settings in this population. The successful refinement and implementation of the protocol could provide a framework to help other scientists leverage sensor technologies in the older adult population. A growing number of clinical trials will incorporate sensor-based wearables in the next decade [23], and our protocol describes how to field-test these devices among the patient population before initiating a clinical trial.

Technology-based interventions hold great promise in the field of fall prevention. Fall detection and medical alert devices can collect and transmit health information about older adults in a timely fashion directly to investigators or clinicians. This data could be used to help researchers and clinicians prevent future falls. However, current studies assessing the feasibility, acceptability, and usability of the Apple Watch in the population most at risk for falls are lacking. Although the Apple Watch is already available to consumers for recreational use, modifications to the settings and app creation are necessary to collect data securely from users and ensure optimal use by older individuals. The GAPcare II protocol may be useful to help researchers who are planning to recruit patients during the COVID-19 pandemic who may be confined to their homes. Our protocol describes how participants can be recruited and can contribute valuable information about cognition and motor skills by using an app from home. While there are a growing number of clinical trials using sensor-based technology, best practices for deploying these technologies among older adults have not yet been established. This field test may help guide the successful integration of wearable devices into clinical studies in this population.

The Apple Watch’s multifunctionality is useful because it combines acceleration-based fall detection methods with caregiver and medical personnel communication. Traditional fall monitoring technology often requires manual activation of a button, which is impossible for a person who may be unable to get up independently after a fall or could be unconscious, and many older adults feel a stigma is associated with wearing a device that is solely for fall alerts. The Apple Watch and other fitness trackers with functions other than fall alerts may have greater appeal for older adults who are seeking to improve their health and stay in communication with loved ones.

While digital health technologies such as wearables show promise, they are expensive and may not be accessible to all older adults. One study conducted in a nationally representative sample of adults in the United States found that patients with low health literacy are less likely to use digital health tools. Conversely, the same study showed that adults with adequate health literacy are more likely to use wearable technologies such as activity trackers, and are more likely to report finding them useful and easy to use [24]. These findings suggest a need to improve usability and functionality of these technologies for all older adults, especially those with low health literacy. Moreover, racial and ethnic minorities use health-related technology less than White older adults, and older adults with higher incomes and greater educational attainment use health-related technology more frequently [25-27]. However, some Medicare Advantage companies are now subsidizing the cost of devices to encourage fitness, which could make these wearables more accessible [28]. To ensure equitable access to wearable technology, other payors should consider subsidizing the cost of these devices that may otherwise be financially inaccessible to many people nationwide. Wearable technology remains inaccessible to many older adults, and efforts to broaden technology literacy and increase cost-effectiveness is of vital concern.

For all older adults, internet and wearable technologies may lack appeal due to concerns about privacy and safety [29], limiting the potential scope and use of Apple Watch technology. Furthermore, there is resistance to uptake and long-term use of wearable technology for fall detection and medical alert in the older adult population, with studies showing that even if a user had an overall positive first impression of the device, it did not lead to long-term device use [30]. The Apple Watch, as well as other Apple products, may appeal more to a younger audience, which may reduce uptake and long-term viability in older populations. For this reason, users need to be educated on the benefits of the maintenance of device use and must receive training to build their internal motivation for device use. Our training protocol and manual could be useful for this purpose. Although drawbacks exist to wearable technology, home-based care is of rising importance, and this technology may improve self-sufficiency among older adults, while also providing important research data to help prevent falls.

### Limitations

While GAPcare II may provide important insights into older adults and their technology preferences, there are several limitations. The sample size of 30 participants is not powered or designed to detect cognitive and motor functioning trajectories and the accuracy of the Apple Watch fall algorithm, but rather to field-test the use of these devices in older adults. Because this study will be conducted in an urban setting among English-speaking ED patients, study results may not be generalizable to rural populations or adults outside of New England. Bilingual research staff are unavailable to conduct recruitment, follow-up, and final interviews; however, expansion among non-English speakers will be paramount in future work to ensure a diverse participant pool. Apple products, including the Apple Watch and iPhone 7, may be cost-prohibitive for patients that represent marginalized populations. We provide iPhones on loan in our study to overcome this barrier. The Apple Watch does not integrate with non-Apple products, which may be an additional challenge individuals need to overcome as they learn how to use these devices for health purposes.

### Conclusions

GAPcare II will provide insights into the feasibility, acceptability, and usability of the Apple Watch, iPhone, and the RIFitTest app in older adults who seek care for falls. Mobile technology could be used as part of clinical trials to objectively measure fall outcomes and could provide detailed continuous information on cognitive and motor functioning without the need for guided assessments by in-person research staff. In the future, wearables could be used as a part of fall prevention interventions to prevent injury before it occurs.

## Appendix

Multimedia Appendix 1 Instructional manual given to participants.

## Abbreviations

ED: emergency department
GAPcare: Geriatric Acute and Post-Acute Fall Prevention Intervention
HIPAA: Health Insurance Portability and Accountability Act
iOS: iPhone operating system
MSTAR: Medical Student Training in Aging Research
NIA: National Institute on Aging
REDCap: Research Electronic Data Capture
RIFitTest: Rhode Island FitTest
RIH: Rhode Island Hospital
TAM: Technology Acceptance Model
TMH: The Miriam Hospital
TPB: Theory of Planned Behavior
UTAUT: Unified Theory of Acceptance and Use of Technology
