# Supplementary material for: The Geriatric Acute and Post-Acute Fall Prevention Intervention (GAPcare) II to Assess the Use of the Apple Watch in Older Emergency Department Patients With Falls: Protocol for a Mixed Methods Study
Source: JMIR Res Protoc. 2021 Apr 1;10(4):e24455. doi: 10.2196/24455 (PMC8050745; doi:10.2196/24455)
Supplement: Multimedia Appendix 1 [file resprot_v10i4e24455_app1.pdf]

GAPcare II Study

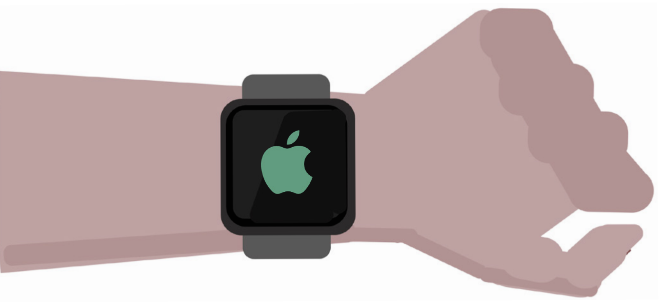

Apple Watch and iPhone Training Manual

ABOUT

What is the GAPcare II Study?

The **GAPcare II Study** aims to provide participants with an Apple Watch to help assess fitness, gait and monitor falls. The overall purpose is to provide patients the best care possible after a fall.

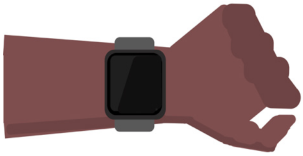

Wear Apple Watch for 30 days

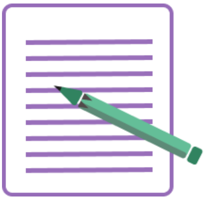

Complete health-based and fall surveys

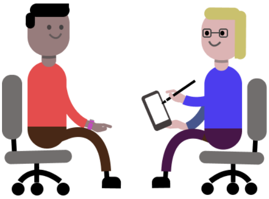

Interview with GAPcare Team on Day 30

GAPcare Study Team
for Additional Questions!
(401) 793-2546

TABLE OF CONTENTS

Apple Watch Setup + Features 2

iPhone Setup + Features 4

Active Tasks

- Reaction Time 7
- Trail Making Test 7
- Gait + Balance 7
- Stroop Test 8
- Timed Walk 8

Fall Diary 9

Digital Health Features of Apple Watch 10

Compensation 12

Study Conclusion + Watch Retrieval 13

Frequently Asked Questions 14

APPLE WATCH SETUP + FEATURES

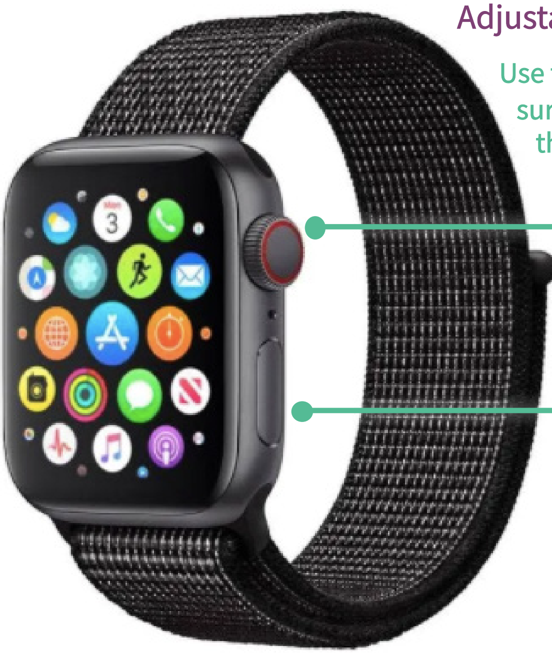

Adjustable Velcro Strap

Use the Velcro strap to make sure the watch is snug to the wrist.

Digital Crown/Scrolling

Click once to enter into the Apple Watch. Zoom in and out of icons using the dial.

Power Button

Hold down for 3 seconds to power on/off.

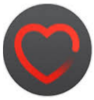

HEART RATE

Click this icon to view your heart rate. If the watch is tight enough to wrist, it should measure.

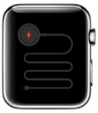

LOW BATTERY

We recommend you charge at dinner time every day.

1 Attach magnetic charging disc to the back of watch face.

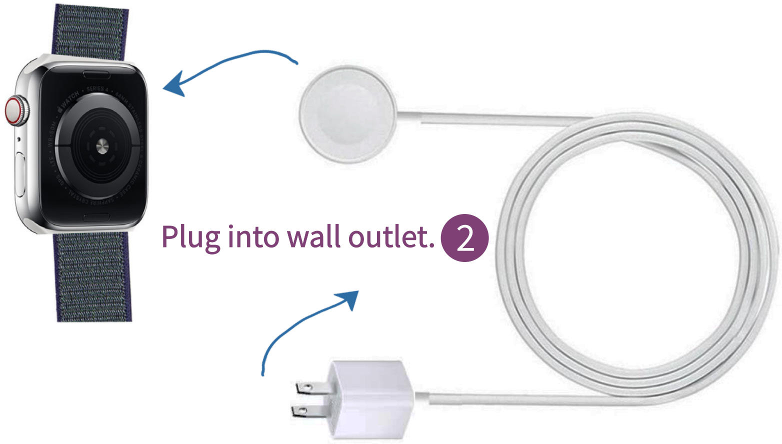

Plug into wall outlet. 2

3 Charge for 1 hour each day.

COMMON FAQs

How often do I wear it? Can I wear the Apple Watch to sleep?

A: We encourage you to wear the watch as much as possible, even during sleep, EVERYDAY, with a minimum of 1 hour charging per day.

Is it ok to wear the watch in the shower?

A: The Apple Watch water resistant and is safe to wear in the shower or while swimming.

How do I get the watch back to you?

A: That part we'll handle. We'll arrange a pickup date and time to retrieve the Apple Watch from your front door.

iPHONE SETUP + FEATURES

Hold Button
Switches “vibrate mode” on/off.

Volume
Use up/down buttons to adjust accordingly.

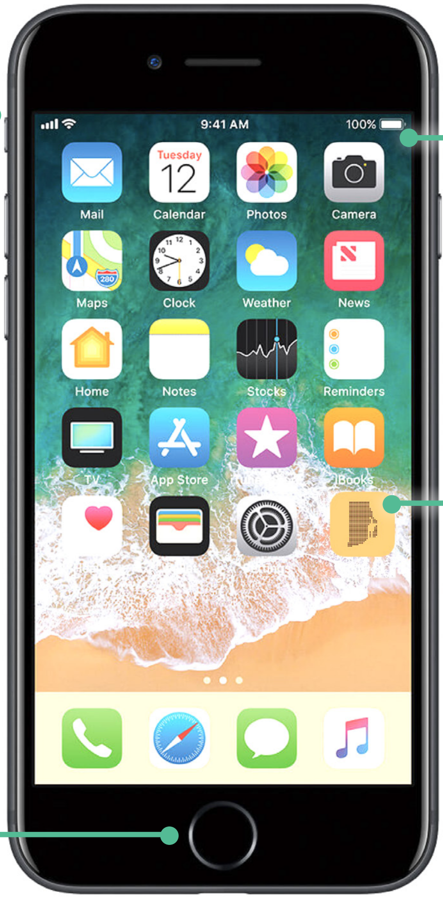

Home Button
Click or push down fingerprint to open phone.

Battery Percent
Bar indicates battery fullness.

Power on/off
Hold down for 3 seconds to power on/off.

RIFitTest Study App
This is where all your surveys and tasks will be.

TOUCH ID
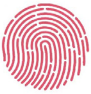
You will have the option to program your fingerprint to unlock the phone.

SWIPE DOWN from the top middle of the screen to display the following features:

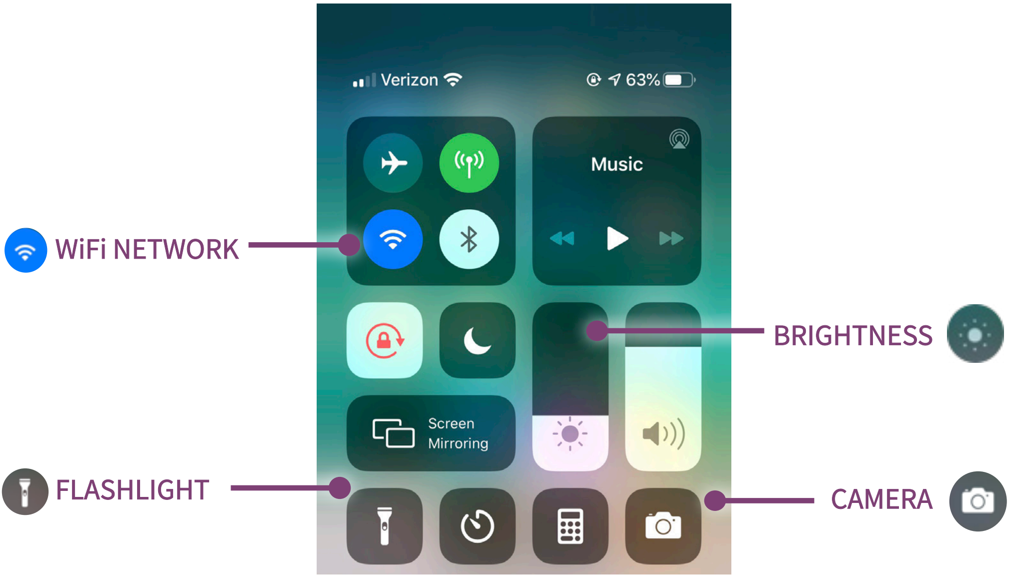

COMMON FAQs

Do I have to have my iPhone on me at all times?

A: Yes, whenever you're wearing the watch, the iPhone needs to be in "radius" or the same room to transmit data, up to about 30-50 feet. We recommend keeping it in your pocket or bag when going out.

How will I know when to complete surveys on the phone?

A: See the survey schedule to know when to complete surveys. You will complete a fall diary daily. You will complete "Active Tasks" once a week.

ACTIVE TASKS

The following 5 tasks should be completed on a weekly basis. The assessments should be helpful in gauging health and memory after a fall.

How OFTEN to complete the Active Tasks?

A: **Weekly**! On **days 1, 7, 14, 21, 28.**
We'll set up reminders for you.

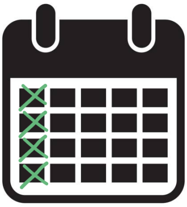

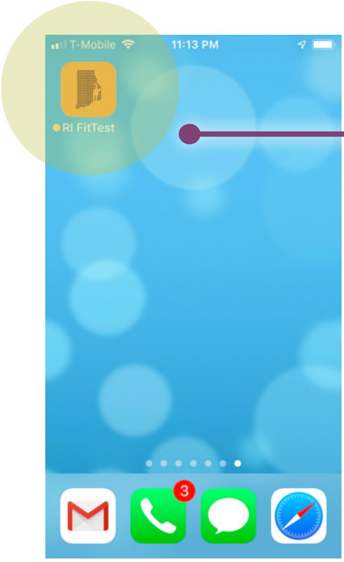

You'll find the ACTIVE TASKS under the RI Fit Test Study App.

REACTION TIME 1

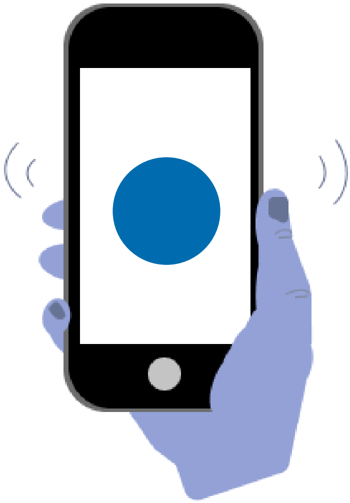

Quickly shake the phone when the blue dot appears on the screen. Repeat several times.

TRAIL-MAKING TEST 2

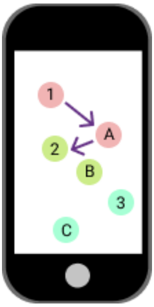

Connect the circles in an alpha-numeric sequence until finished. (1 ... A ... 2 ... B)

TIMED WALK 3

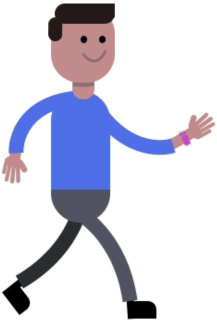

Walk about 109 yards at a swift pace until the app indicates to stop. It's best to do this activity outside safely if possible.

STROOP TEST 4

Click the name of the font color shown, not the color as written. Complete this several times.

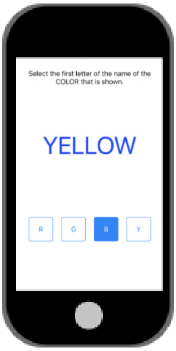

Since, "Yellow" is written in blue font, "B" for Blue is correct.

GAIT + BALANCE 5

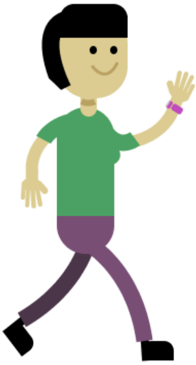

1. Place the iPhone in your pocket and walk about 20 steps in a straight line.
2. Turn around and walk back to where you started.
3. Stand still for 30 seconds.

For an additional help or instructions with Active Tasks, please call the GAPcare Team at (401) 793-2546

FALL DIARY

Everyday you should complete the fall diary, which can be found in the RIFitTest App. This helps us to know if the Apple Watch is working correctly.

Click into the RIFitTest Study App, and click “On Demand” Surveys.

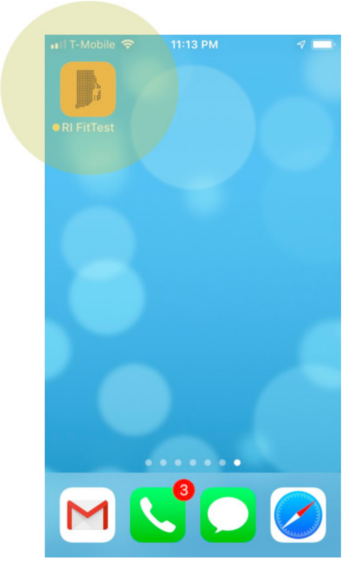

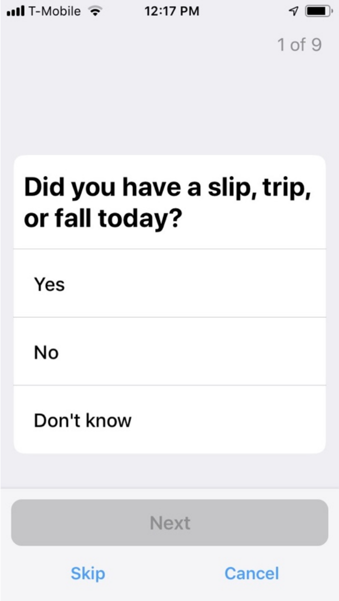

Indicate if you sustained an injury!

APPLE WATCH: **DIGITAL HEALTH FEATURES**

The Apple Watch has a number of health features, some of which are still being discovered. Only **Fall Detection** will be a necessary feature of the study, but feel free to explore other ones!

FALL DETECTION

In the event of a fall, the Apple Watch has acceleration-based tracking, sensing that the body has come to a "hard fall" to the ground.

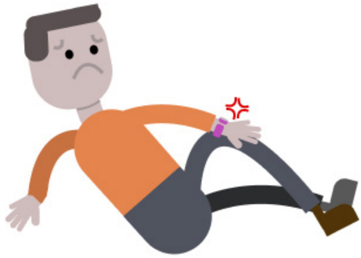

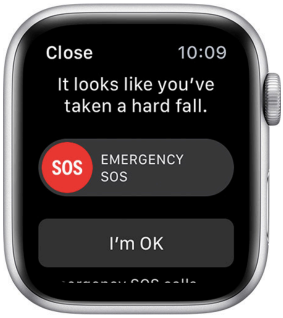

If detected, you'll have the option of dismissing the alert or dialing emergency services yourself.

MOTIONLESSNESS for greater than a minute will result in an automatic dial-out to emergency services.

i Previous fall detection devices only functioned with press buttons. This solves the issue of losing consciousness and being unable to dial out!

A healthy resting heart rate is anywhere from 60 - 100 bpm. A bit higher or lower than this range is also normal!

HEART RATE

The Apple Watch can track your heart rate by measuring the pulse on your wrist:

After 5 seconds of measuring, the watch can tell you:

- Resting Heart Rate
- Walking Heart Rate

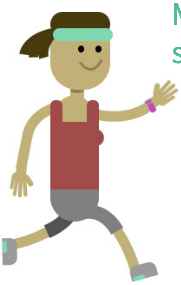

Measure health trends, such as:

- Step count
- Calories burned
- Hours stood

ACTIVITY

Check your step count, calories burned, and overall activity for the day.

Electrocardiogram (ECG)

Through the ECG app: Check your heart rhythm, measured as an ECG, by holding on the Digital Crown for 1 minute.

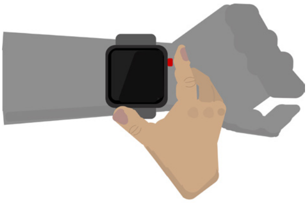

SLEEP

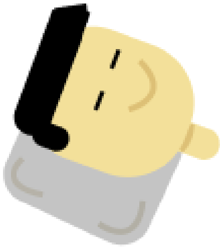

Check the hours of sleep you get, time spent in REM, etc. A number of apps including “AutoSleep” and “Pillow” are available!

COMPENSATION

By the end of the study, participants receive a total of $65, after the Apple Watch is returned.

Study Conclusion + Device Return: $50
End of Study Interview: $15

$65

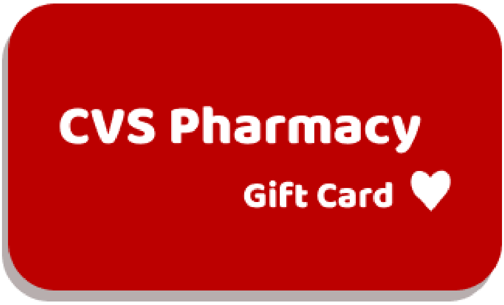

Please allow up to 24 hours for the card to fill.

STUDY CONCLUSION + WATCH RETRIEVAL

Your time in the GAPcare Study will last 30 days, or approximately 4 weeks. At the end of the 4 weeks, there will be a number of steps to follow:

1. There will be a 30-minute interview with you about your time in the study and experience with the Apple Watch. This will be done over video chat or phone call.

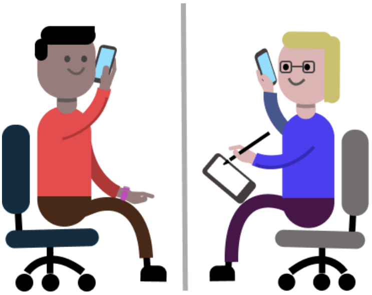

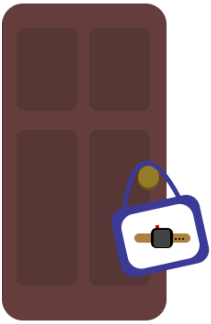

2. After, we'll schedule a time to pick up the Apple Watch/iPhone from your front door or mailbox, no contact necessary. You'll hang up the bag at a prespecified time.

FREQUENTLY ASKED QUESTIONS:

How long will my study participation last?

The study period is 30 days (about 4 weeks).

Who is in charge of the study?

Dr. Elizabeth Goldberg, an emergency room physician at Rhode Island and Miriam Hospitals.

How many people are doing this study?

We're looking to recruit 30 patients aged 65 or older and/or their caregivers for this study.

Who runs this study? Brown? Lifespan?

Good question. The GAPcare Study is run by a Brown affiliated physician and students. Lifespan Emergency Medicine Clinical Research is also part of the team. It's funded by the NIH and Apple.

I forgot to wear the watch for a day.. what happens now?

No problem! Just wear it as much as possible.

Can I wear the watch in the shower? To sleep?

Yes and yes! Falls occur frequently during these times, so we encourage you to wear it. The watch only needs to charge for an hour a day.

I completely missed my time to do the Active Tasks this week, what should I do now?

No problem! Just complete them as soon as possible.

My fingerprint stopped working for the phone . . . how do I get in?

There should be a backup passcode to get into the phone. As always, please call with difficulties!

Health data is private . . . is everyone going to have access to my fall and watch data?

The study team has access during the 30-day period, and all of your data is assigned to a study number rather than your name. Apple may have access to deidentified study data for quality control purposes.

I forgot to log a fall in the Fall Diary..

That's okay! Just log it as soon as possible.

How do I know that the Apple Watch is clean? Especially in the COVID-19 era, I'm nervous..

We quarantine the device after use and follow hospital approved disinfecting procedures.

Thank you so much for your participation! We hope you enjoyed the study. Please call with any comments or questions!

- GAPcare Team: (401) 793-2546
